# High-throughput strategies for penicillin G acylase production in *rE. coli* fed-batch cultivations

**DOI:** 10.1186/1472-6750-14-6

**Published:** 2014-01-21

**Authors:** Ana Maria Vélez, Adilson José da Silva, Antonio Carlos Luperni Horta, Cintia Regina Sargo, Gilson Campani, Gabriel Gonçalves Silva, Raquel de Lima Camargo Giordano, Teresa Cristina Zangirolami

**Affiliations:** 1 Chemical Engineering Department, Federal University of São Carlos (UFSCar), Rodovia Washington Luís, km 235, C.P. 676, CEP 13565-905 São Carlos, SP, Brazil; 2 Institute of Chemistry, Federal University of Goiás, Campus Samambaia, C.P. 131, CEP 74001-970 Goiânia, GO, Brazil

**Keywords:** Penicillin G acylase, Recombinant *E. coli*, Auto-induction medium, Fed-batch culture

## Abstract

**Background:**

Penicillin G acylase (PGA) is used industrially to catalyze the hydrolysis of penicillin G to obtain 6-aminopenicillanic acid. In *Escherichia coli,* the most-studied microorganism for PGA production, this enzyme accumulates in the periplasmic cell space, and temperature plays an important role in the correct synthesis of its subunits.

**Results:**

This work investigates the influence of medium composition, cultivation strategy, and temperature on PGA production by recombinant *E. coli* cells. Shake flask cultures carried out using induction temperatures ranging from 18 to 28°C revealed that the specific enzyme activity achieved at 20°C (3000 IU gDCW^-1^) was 6-fold higher than the value obtained at 28°C. Auto-induction and high cell density fed-batch bioreactor cultures were performed using the selected induction temperature, with both defined and complex media, and IPTG and lactose as inducers. Final biomass concentrations of 100 and 120 gDCW L^-1^, and maximum enzyme productivities of 7800 and 5556 IU L^-1^ h^-1^, were achieved for high cell density cultures using complex and defined media, respectively.

**Conclusions:**

To the best of our knowledge, the volumetric enzyme activity and productivity values achieved using the complex medium are the highest ever reported for PGA production using *E. coli*. Overall PGA recovery yields of 64 and 72% after purification were achieved for crude extracts obtained from cells cultivated in defined and complex media, respectively. The complex medium was the most cost-effective for PGA production, and could be used in both high cell density and straightforward auto-induction protocols.

## Background

Penicillin G acylase (PGA) is used industrially to catalyze the hydrolysis of penicillin G to obtain 6-aminopenicillanic acid (6-APA), which is a key intermediate for the synthesis of β-lactam antibiotics [1]. These drugs account for the largest fraction of global sales of antibiotics, and comprised 60% of the 5 × 10^7^ kg/year produced worldwide in 2003 [1]. The production of semi-synthetic β-lactam antibiotics requires ever-increasing quantities of PGA, and the annual consumption of this enzyme is estimated to be in the range of 10–30 million tons [2]. The unmet demand for PGA at an acceptable cost could be supplied by improving the processes used for its production.

PGA can be produced by a variety of microorganisms, including bacteria, fungi and yeasts. The microorganism that has been most widely studied for this purpose is *Escherichia coli*, which accumulates the enzyme in the periplasmic cell space. The enzyme precursor is synthesized as an inactive 96 kDa pre-pro-PA, which contains a signal peptide at its N terminus [3] that mediates the translocation of pro-PA into the periplasm. This intermediate is further processed into α (23 kDa) and β (63 kDa) PGA chains during autoproteolytic reactions [4].

In the past, PGA has mainly been manufactured by fed-batch as well as by batch processes, using recombinant *E. coli*, *Bacillus megaterium*, or *Arthrobacter viscosus*, and sucrose or glucose as carbohydrate substrates [5].

Optimization of the performance of cultures for recombinant enzyme production has two main objectives: high-level gene expression, and intense formation of biomass containing a high activity level of soluble, correctly-folded intracellular enzyme [6].

Strategies for high-level gene expression involve increasing the efficiency of one or more gene expression steps [3]. In the case of the *pac* gene, encoding penicillin G acylase of *E. coli*, *B. megaterium* and other organisms [7,8], many different procedures have been employed to this end. The efficiency of transcription of the *pac* gene can be genetically modulated by mutation or removal of the regulatory region, mutations in the transcription initiation region, or by replacement of the *pac* promoter [9-11]. The post-translational processes, which are crucial for obtaining soluble active PGA, have been investigated to identify optimum host/vector combinations that can efficiently produce the mature protein [3,6], with co-expression of helper proteins that assist the formation of correctly-folded active PGA [6,12] . In addition, cloning the *pac* gene from different microorganisms in *E. coli* is another strategy that has been used to improve enzyme production [7,13,14].

Temperature plays a very important role in modulating the expression of key genes involved in recombinant protein production [15-17]. The rate of protein synthesis is reduced at low temperatures, which increases the formation of correctly-folded biomolecules [18]. Selection of a suitable temperature is also crucial to ensure that a balanced flux is maintained throughout the stages of protein synthesis (transcription) and maturation (translocation and periplasmic processing), in order to avoid accumulation of immature precursors and optimize expression of the *pac* gene [10]. Otherwise, the formation of insoluble protein aggregates (inclusion bodies) can occur [19]. Keilmann et al. [18] also reported that the initiation step of translation of the *pac* mRNA is blocked at high temperatures.

The production of PGA in bioreactor cultivations has been widely studied using both batch [20-22] and high cell density fed-batch cultures [6,23,24]. Fed-batch cultivation is considered to be the most efficient process for the production of inducible heterologous proteins, including PGA, because the nutrient supply can be modulated to firstly achieve high biomass formation and then proceed with the induction [25]. However, performing a high cell density culture (HCDC) is a challenge, mainly because control of both the dissolved oxygen concentration and the flow rate of the feed medium is problematic at high biomass concentrations [24,26].

The auto-induction approach [27,28] offers an alternative to fed-batch cultures. Biomass concentrations of up to 40 gDCW L^-1^[29,30] can be obtained directly from batch experiments employing a mixture of glucose, glycerol, and lactose, with minimal requirements for handling of the expression culture [31]. However, this strategy has not yet been reported for PGA production in shake flask or bioreactor cultures of recombinant *E. coli*.

The composition of the culture medium is another important consideration in design of a suitable cultivation strategy, because it not only affects cell growth, but also influences gene expression and, consequently, the pool of proteins accumulated in the cells. Both defined and complex media have been employed for PGA production, but no reported studies have addressed the influence of medium composition on the performance of processes used for PGA recovery from cell lysates. In addition, the composition of the medium, as well as the induction strategy (especially the inducer selected), play important roles in the post-induction cell response. So far, the majority of published works have focused on the use of isopropyl-β-D-thiogalactopyranoside (IPTG) as the inducer [6,21,22,24]. Nonetheless, the fact that IPTG is an expensive and potentially toxic chemical restricts its application as an inducer in the industrial production of recombinant proteins. Lactose, on the other hand, is an inexpensive, natural, environmentally friendly inducer that can be used in expression systems based on the *lac* promoter. Despite these advantages, only one study has so far considered PGA production induced by lactose [23].

With the goal of developing an optimized protocol for PGA production, the main objectives of the present study were therefore to: i) assess the influence of induction phase temperature on PGA production; ii) compare auto-induction and conventional fed-batch cultivation strategies for PGA production in a bioreactor; and iii) analyze the influence of the culture medium composition and inducers on protein synthesis and purification.

## Results

### Influence of temperature on PGA production

**Figure 1:**
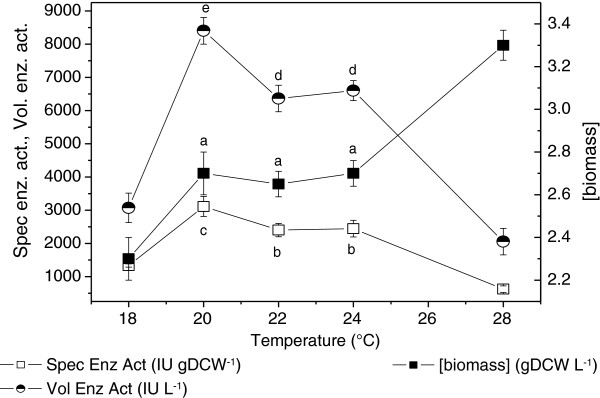
**Effect of induction temperature upon specific activity (Eq. 4) and volumetric enzyme activity (Eq. 5) of PGA produced by recombinant *E. coli *grown in shake flasks containing LB medium after 24 h of induction.** All experiments induced with 0.25 mM IPTG, T_growth_ = 37°C, OD_induction_ = 1.5. Error bars for specific enzymatic activity, volumetric enzyme activity and biomass concentration are standard deviations from triplicates. Means with different letters (a–e) differ significantly according to the Tukey’s test (p <0.05).

As already stated above, temperature plays an important role in PGA synthesis by *E. coli* cells, and values between 22 and 37°C have been reported for the induction phase [7,32]. Here, induction temperatures ranging from 18 to 28°C were tested in the shake flask cultures. Figure 1 shows the specific and volumetric enzyme activities after 24 h of induction at different temperatures. The specific enzyme activity was ~3000 IU gDCW^-1^ at 20°C, ~2500 IU gDCW^-1^ at 22 and 24°C, and decreased to less than 1000 IU gDCW^-1^ at 28°C. As expected, the final biomass concentration increased at higher temperatures, ranging from 2.3 (at 18°C) to 3.3 gDCW L^-1^ (at 28°C).

Despite the reduced biomass formation observed at lower temperatures, the enzyme concentration per volume of culture medium was significantly improved at 20°C. The enzyme production achieved at this temperature was 2 to 3-fold superior to values reported in the literature [6,23,33], probably due to the temperature employed during the induction phase in the earlier studies, which ranged from 28 to 30°C. Plasmid stability was also favored at 20°C, with values of 70-80% obtained after 24 h of induction. When a temperature of 24°C was used, plasmid retention decreased to 53% at the end of the induction period.

**Figure 2:**
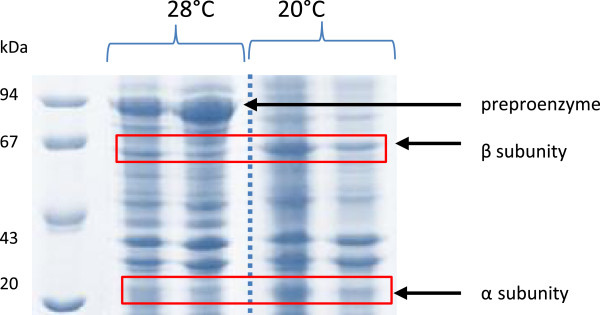
**SDS-PAGE of cell lysates for recombinant *E. coli *grown in shake flasks containing LB medium after 24 h of induction.** Experiments induced with 0.25 mM IPTG. (1) protein standards; (2) insoluble proteins (28°C); (3) soluble proteins (28°C) (4) insoluble proteins (20°C); (5) soluble proteins (20°C).

These results pointed out 20°C as the best temperature for PGA production by r*E. coli*. This value is lower than the optimum temperature (28°C and 22°C) identified by Cheng et al. and Dai et al. [7,32], respectively, for maximizing PGA production with IPTG as inducer. In these studies, constructs were based on plasmids pET24a and pMLB1023, whereas in the present work, plasmid pT101/D-TOPO was used. So, the results suggest that the optimum temperature for PGA synthesis may also depend on the construct (plasmid and gene). Further evidence of the strong influence of temperature on PGA synthesis was obtained from the SDS-PAGE analyses of disrupted cells after 24 h of induction at 28 and 20°C (Figure 2). An intense band corresponding to pre-pro-enzyme (~90 kDa) was found for the cells induced at 28°C, for both soluble and insoluble fractions (lanes 2 and 3). The formation of large amounts of pre-secretory product could be due to overloading of the transportation machinery of the cell. As a result, untranslocated pre-pro-enzyme may have accumulated and formed aggregates of an inactive form of the enzyme [21]. On the other hand, when the induction took place at 20°C, subunits α and β were observed (lanes 4 and 5), but the pre-pro-enzyme was not present.

### Growth and protein production in bioreactor culture using the auto-induction strategy

Before investigating an induction strategy for high cell density cultures (HCDC) based on a low induction temperature, the alternative auto-induction protocol was used to perform bioreactor experiment B1 and validate the shake flask results. Despite being frequently used for protein production using T7-based *E. coli* expression systems in shake flask cultures [27,34], only a few investigations have considered bioreactor cultures based on auto-induction complex medium formulations [29,30,35].

**Figure 3:**
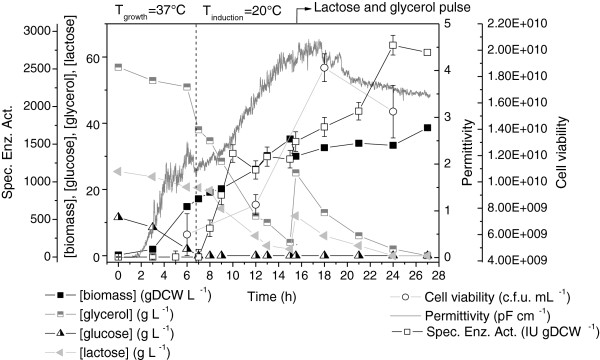
**Growth, carbon sources consumption and PGA production by recombinant *E. coli *in intermittent fed-batch culture B1 with auto-induction medium.** T_growth_ = 37°C and T_induction_ =20°C. Pulse of glycerol and lactose added after 15.5 h of culture. Error bars for cell viability and specific enzymatic activity are standard deviations from triplicates.

Figure 3 shows the main results obtained for the auto-induction culture. Intense biomass formation was observed in the first 6 h of culture (μ_max_ = 0.73 h^-1^), assisted by the high growth temperature (37°C) and fueled by the consumption of glucose and proteins/amino acids from the yeast extract and tryptone (components of the complex medium). Glucose prevents the uptake of lactose, and its exhaustion (at ~6.5 h) signaled the beginning (t_induction_ = 0 h) of heterologous protein synthesis induced by the lactose present in the medium to induce expression [27]. Shortly after glucose depletion, the temperature was reduced to 20°C to reproduce the induction strategy selected from the shake flask results. The PGA specific activity data (Figure 3) were indicative of fast enzyme accumulation in the cells, reaching 2800 IU gDCW^-1^ after 17.5 h of induction. This value was similar to the maximum specific activity observed in the flask experiments (Figure 1) carried out at the same temperature (20°C).

The biomass concentration increased continuously up to 36 gDCW L^-1^, with a moderate growth rate (μ = ~ 0.09 h^-1^) after the temperature decrease (between 7 and 15 h), and a slow growth rate (μ = ~ 0.02 h^-1^) during the last 12 h of induction due to intensification of the metabolic burden related to protein expression [36-38]. The permittivity signal (Figure 3) closely followed the biomass formation profile up to ~16 h of culture. The capacitance sensor measures the dielectric permittivity produced by a polarized cell membrane when it is subjected to low radio frequencies. Since this only occurs for viable cells [39], the shift in the permittivity curve reflected a decrease in the viable cell concentration due to the stress associated with protein production, as also shown by the c.f.u. counts [36]. The measured loss of viability was also influenced by the decrease in the proportion of cells retaining the plasmid, which changed from 90% (at 5.5-11.5 h of induction) to 85% (at 17.5 h of induction).

The scale-up auto-induction medium composition proved to be effective. The high initial glucose concentration prevented early protein expression, but did not cause formation of high levels of inhibitory metabolites: the concentrations of acetic, formic, and lactic acids remained below 2 g L^-1^ throughout the cultivation [40,41]. The low formation of organic acids was assisted by the choice of glycerol as the main carbon source. Together with glucose released by lactose hydrolysis due to β-galactosidase, glycerol was steadily consumed and both carbon sources were used by the cells to support growth and protein production during the entire induction phase. Galactose, which is not assimilated by *E. coli* BL21(DE3) cells, accumulated in the culture medium (data not shown). At 15.5 h of cultivation, an additional pulse of a solution containing lactose and glycerol was added in order to avoid limitation of protein synthesis due to a lack of either carbon source or inducer. Use of this simple cultivation strategy yielded a bulk PGA volumetric enzyme activity exceeding 93775 IU L^-1^ within 17.5 h of induction, corresponding to a productivity of 3907 IU L^-1^ h^-1^ (or 5358 IU L^-1^ h^-1^, considering the elapsed induction period).

### Growth and protein production for scaled-up high cell density cultures

The lactose-based induction strategy was further scaled up as a high cell density culture (HCDC) using complex medium (culture B2). For comparison, a HCDC using defined medium and IPTG as inducer (culture B3) was also carried out. The feeding media for both fed-batch cultures contained exclusively glycerol as carbon source (Table 1), and were supplied following an exponential profile (Equation 7) based on the actual growth rates, as described below (Methods section). Induction was triggered by pulses of lactose (B2) or IPTG (B3), shortly after the temperature had been reduced to 20°C.

**Table 1:** Composition of media used in bioreactor cultivations

	**Auto-induction**		**Complex media**		**Defined media**	
**Nutrient (g L**^**-1**^**)**	**Batch**	**Pulse**	**Batch**	**Fed-batch**	**Batch**	**Fed-batch**
Glucose	10.0	---	10.0	---	---	---
Glycerol	60.0	258.3	40.0	800	40.0	800.0
Lactose	20	130.0	---	---	---	---
Tryptone	10.0	10.0	10.0	10.0	---	---
Yeast extract	5.0	5.0	5.0	5.0	---	---
MgSO_4_.7H_2_O	0.5	0.5	0.5	40.0	1.6	20.0
KH_2_PO_4_	3.4	3.4	3.4	3.4	17.7	21.28
(NH_4_)_2_HPO_4_	---	---	---	---	5.3	6.4
Na_2_HPO_4_.12H_2_O	9.0	9.0	9.0	9.0	---	---
Citric acid	---	---	---	---	2.27	2.27
NH_4_Cl	2.7	2.7	2.7	2.7	---	---
Na_2_SO_4_	0.7	0.7	0.7	0.7	---	---
Thiamine	---	---	---	---	45.0	45.0

Auto-induction medium: cultivation B1; complex medium: cultivation B2; defined medium: cultivation B3.

**Figure 4:**
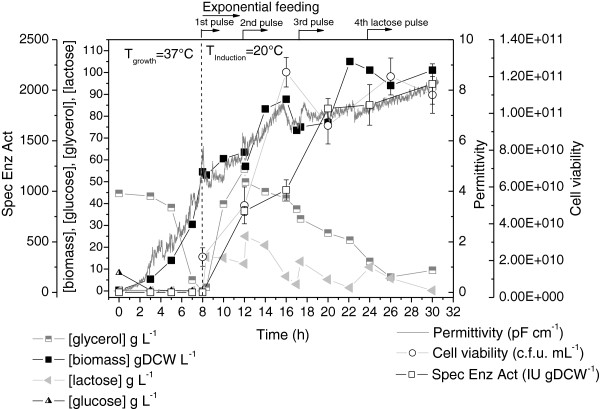
**Growth, carbon sources consumption and PGA production by recombinant *E. coli *using fed-batch culture B2 with complex medium.** T_growth_ = 37°C and T_induction_ = 20°C. Lactose pulses added at 8, 12, 17 and 24 h. Feeding started at 7.3 h. Error bars for cell viability and specific enzymatic activity are standard deviations from triplicates.

The results obtained for the complex medium fed-batch culture (B2) are shown in Figure 4. Due to the low solubility of lactose and the increasing biomass concentration, the inducer was supplied in the form of multiple 400 mL pulses of solutions containing 200 g L^-1^ lactose, which were added after every 20 gDCW L^-1^ (~40 OD_600nm_ units) increase in the cell concentration. This strategy was based on the results obtained in the previous auto-induction culture (B1), relating biomass formation to lactose uptake (Figure 3). Lactose concentration measures during cultivation B2 showed that, despite continuous hydrolysis of lactose, it was possible to maintain a residual inducer concentration capable of promoting *pac* gene expression throughout the induction phase.

The cultivation strategy adopted was effective for both biomass formation and enzyme production. The initial growth phase was carried out at 37°C, lasted 7.3 h, and led to a biomass formation of 33 gDCW L^-1^ (μ_max_ = 0.68 h^-1^). The continuous fed-batch phase was then started, and supported continuous cell accumulation up to 105 gDCW L^-1^ during the next 14.8 h, with high viability, as confirmed by the permittivity data and the c.f.u counts. Enzyme production started after the first pulse addition (at 8 h of culture), at a biomass concentration of 54 gDCW L^-1^, and the specific enzyme activity rapidly increased to 1800 IU gDCW^-1^ within the first 12 h of induction, with complete hydrolysis of the lactose supplied. During the last 10 h of induction, the enzyme synthesis rate slowed, and the specific enzyme activity reached a final value of 2061 IU gDCW^-1^. This shift in the enzyme production profile was mainly due to intensified stress associated with protein synthesis [36], which caused a reduction in plasmid retention from 97% (at 4-8 h of induction) to 89% (at 12 h of induction). Once again, the formation of inhibitory metabolites was negligible (2.1 g L^-1^ acetate), despite glycerol accumulation during the first 4 h of feeding [40,41]. It is also important to clarify that the fluctuations in cell concentration and viability were caused by the withdrawal of large volumes of culture medium (~400 mL) prior to the addition of the lactose pulses, in order to ensure that the reactor volume remained within the operational range.

In summary, the lactose-based complex medium fed-batch strategy yielded a PGA volumetric enzyme activity equivalent to 208222 IU L^-1^ within 24 h of induction, which corresponded to a productivity of 6941 IU L^-1^ h^-1^ (or 9465 IU L^-1^ h^-1^, considering the elapsed induction period). These values are about twice those observed for the B1 auto-induction culture, because the biomass formation phase was extended in the continuous fed-batch operation mode and occurred simultaneously with intense protein production during the first 15 h of induction, due to the low induction temperature employed.

**Figure 5:**
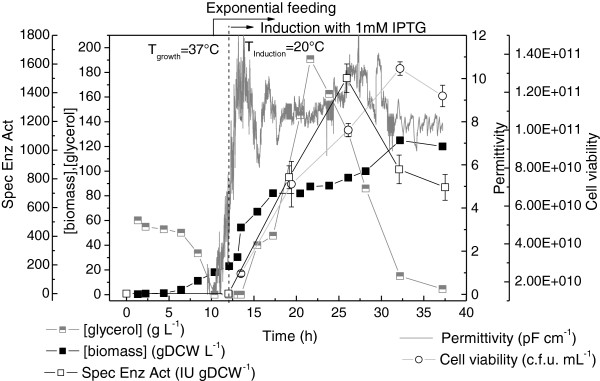
**Growth, glycerol consumption and PGA production by recombinant *E. coli *in fed-batch culture B3 with defined medium.** T_growth_ = 37°C and T_induction_ =20°C. Induction by IPTG pulse at t = 13 h. Feeding started at 10.1 h. Error bars for cell viability and specific enzymatic activity are standard deviations from triplicates.

The influence of inducer and medium composition on PGA production and purification was assessed using continuous fed-batch cultivation B3 with defined medium and IPTG as inducer. The main results are presented in Figure 5. Glycerol was the only carbon source during the initial batch and fed-batch phases. The feed supply was initiated 10 h after the beginning of the culture, as soon as the glycerol from the batch culture was exhausted, yielding a biomass concentration of 18 gDCW L^-1^ and a maximum specific growth rate of 0.45 h^-1^. The temperature was then reduced from 37 to 20°C, and a pulse of IPTG was applied at 13 h (at a biomass concentration of 54 gDCW L^-1^) to initiate the induction phase. Despite the temperature reduction, the cells continued to grow at a moderate rate (~ 0.1 h^-1^), reaching 82 gDCW L^-1^ within 4 h of induction.

The B2 and B3 continuous fed-batch cultures showed equivalent performance (Figures 4 and 5) up to the moment of induction. Obviously, due to the inherently lower biomass yield for the defined medium (Y_X/S_ values of 0.44 and 0.55 g g^-1^ were obtained for the defined and complex media, respectively), in the case of culture B3 a longer growth phase (13 h) was required before the desired biomass concentration was attained (~54 g L^-1^) and the induction phase could be initiated. However, throughout the induction phase, the B3 culture showed fluctuations on the permittivity data and accumulation of glycerol. The instability can be partially attributed to the sudden temperature decrease from 37°C to 20°C prior to IPTG addition. Due to the reduced growth rate imposed at 20°C, glycerol started to accumulate in the broth. Glycerol accumulation was probably even intensified by the salt precipitation observed after the temperature decrease, due to the low aqueous solubility of several of the defined medium components, especially KH_2_PO_4_ (Table 1). In such a situation, growth became limited by a nutrient other than the carbon source. The accumulation of glycerol at concentrations of up to approximately 80 g L^-1^ was tolerated by the cells [29], but inhibition occurred when this value was exceeded, causing stagnation of growth at between 16 and 25 h of cultivation. After feeding was interrupted, the cells started to consume the excess glycerol (and salts) accumulated in the medium, and cell growth was restored at the end of induction phase, leading to a final biomass concentration of 120 gDCW L^-1^. It is also noteworthy that, once again, despite the high concentration of glycerol accumulated in the medium, the concentration of the organic acids that are commonly present did not exceed 2.5 g L^-1^. This is the main advantage of using glycerol as carbon source for HCDC. In contrast to glucose, its uptake by the cells does not trigger overflow metabolism and, as long as adequate aeration conditions are provided, there is no risk of inhibition of growth or protein synthesis due to metabolite formation [42].

The facts described above also affected protein production. The specific enzyme activity increased to a maximum of 1500 IU gDCW^-1^ after 12 h of induction, and subsequently decreased continuously. This activity level was significantly lower than the maximum values observed for experiments B1 and B2 (Figures 3 and 4), as well as for the shake flask cultures performed using the same induction temperature (Figure 1). The gradual decline in the enzyme yield also showed a relationship with plasmid stability, which diminished from 87% (after 5 h of induction) to 75% (after 13 h of induction) and 65% (after 19 h of induction, at the end of the cultivation). Plasmid retention during this experiment was inferior compared to the B1 and B2 cultures carried out with complex medium, which showed retentions of >80% after 18 h of induction. It is known that plasmid stability is generally favored in amino acid-enriched media [43]. Nevertheless, mainly due to the high final biomass concentration achieved, fed-batch cultivation B3 yielded a PGA volumetric enzyme activity equivalent to 89000 IU L^-1^ within 24 h of induction, corresponding to a productivity of 2393 IU L^-1^ h^-1^ (or 3680 IU L^-1^ h^-1^, considering the elapsed induction period). On the other hand, care must be taken before choosing the conventional defined high cell density medium (HDF medium) [42] for heterologous protein production when the synthesis is favored by low temperature.

**Figure 6:**
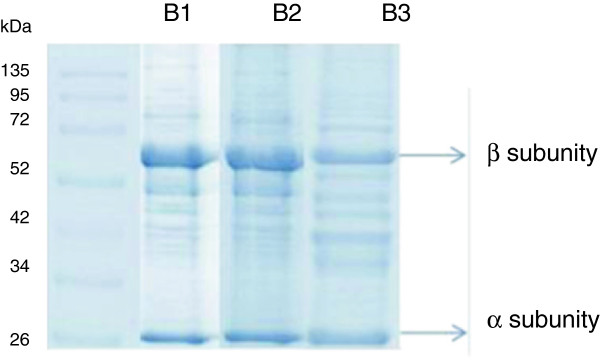
**SDS-PAGE of clarified fraction obtained after lysis of recombinant *E. coli *cells cultivated in different media after 24 h of induction.** B1: auto-induction; B2: Fed-batch with complex medium and B3: Fed-batch with defined medium. All samples contained 1 mg/mL of protein.

The electrophoresis results for the three bioreactor cultures (Figure 6) showed that the fraction corresponding to contaminant proteins present in the enzyme extracts is similar, regardless of either the cultivation strategy or the inducer used. This was due to the low temperature employed during the induction phase (20°C), which helped to reduce the production of endogenous proteins by *E. coli.*

### Partial purification of recombinant PGA from complex and defined media cultivations

Besides identification of the most suitable cultivation strategy for recombinant enzyme production, it is also important to assess the impact of the chosen cultivation protocol on the performance of the purification process. One of the drawbacks often associated with the use of complex medium is possible stimulation of the synthesis of diverse contaminant proteins, which could impair purification of the target protein.

The results obtained for the partial purification of PGA present in crude extracts obtained from cells grown in both types of medium (complex and defined) are shown in Table 2. The clarified extract generated from the cells cultivated in complex medium had a higher protein content, and consequently a lower specific enzyme activity. This extract retained ~30% of the PGA activity initially present, due to saturation of the resin. The recovery of PGA could be improved by reducing the loading of enzyme per gram of resin. Consequently, the global yields for the purification process were 72 and 64% for the extracts derived from the complex and defined media, respectively.

**Table 2:** **Results obtained for the batch adsorption-desorption process of PGA from recombinant *E. coli *** **using the Streamline SP XL resin at pH 4.8**

**Samples**	**Cells harvested from complex medium**			**Cells harvested from defined medium**		
	**Enz. Act. (IU L**^**-1**^**)**	**Prot. Conc. (mg**_**prot **_**L**^**-1**^**)**	**Spec. Act. (IU mg**_**prot**_^**-1**^**)**	**Enz. Act. (IU L**^**-1**^**)**	**Prot. Conc. (mg**_**prot **_**L**^**-1**^**)**	**Spec. Act. (IU mg**_**prot**_^**-1**^**)**
Clarified	500 ± 45	480.8 ± 32.1	1.04 ± 0.70	420 ± 51	305.1 ± 15.6	1.38 ± 0.42
Extract	130 ± 21	213.1 ± 43.4	0.61 ± 0.07	150 ± 32	235.1 ± 43.1	0.64 ± 0.21
Washing	0.2 ± 0.1	3 ± 1	0.3 ± 0.2	0.2 ± 0.1	0.4 ± 0.1	0.1 ± 0.3
Eluate	1200 ± 82	510.6 ± 14.2	2.35 ± 0.03	893 ± 76	231.1 ± 16.2	3.86 ± 0.34
η_act_^el^ (%)	97 ± 2			99 ± 2		
η_act_^ads^ (%)	74 ± 6			64 ± 4		
η^process^ (%)	72 ± 5			64 ± 6		
P.F.	2.3 ± 0.3			2.8 ± 0.4		

The eluted activity yields all exceeded 97%, indicative of similar interactions between the resin and the clarified fractions, irrespective of their source. Furthermore, the salt concentration in the eluate buffer was satisfactory, and almost all the recombinant PGA was detached, leading to the high recovery observed in this step. Overall, 2.3-fold and 2.8-fold purity increases were obtained for the extracts from the complex and defined media cultivations, respectively.

These results are similar to those obtained by Pinotti et al. [44], using the same protocol and resin for PGA purification. Although higher purification factors have been reported for penicillin acylase [45], the main objective of this study was not to optimize the methodology, but rather to compare the performance of the purification process when handling cell lysates derived from cells cultivated and induced under different conditions. The results presented here demonstrate that cultivation in complex medium is a better strategy for PGA production, and does not jeopardize the performance of the purification process.

## Discussion

### Comparison of enzyme production using different cultivation strategies

**Figure 7:**
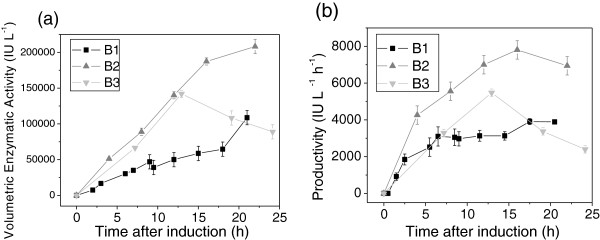
**Profiles of (a) volumetric enzyme activity estimated from Eq. 5 (IU/L) and (b) productivity (Eq. 6) of recombinant PGA in *E. coli *during induction phase.** Error bars are standard deviations from triplicates.

A comparison of all the cultivation strategies, in terms of enzyme production, is provided in Figure 7. As already mentioned, the continuous fed-batch strategy with complex medium (experiment B2) provided the best volumetric enzyme activity and productivity (Figures 7a and 7b). The auto-induction culture (experiment B1) exhibited a stationary protein productivity profile after 6.5 h of induction (Figure 7b), because the biomass concentration remained almost constant, while the specific enzyme activity increased linearly with induction time (Figure 3). Conversely, for the continuous fed-batch cultivation (B2), both biomass concentration and specific PGA activity increased during the first 16 h of induction (Figure 4), resulting in a steeper increase in productivity (Figure 7b). Despite the low induction temperature used, Figure 7b shows that 16 - 18 h of lactose induction was sufficient to achieve high enzyme productivity for both auto-induction (B1) and HCDC (B2) strategies. Figure 7a shows that the initial rate of enzyme accumulation was similar for HCDC cultures B2 and B3 during the first 13 h of induction, due to the increasing biomass concentration and specific enzyme activity (Figures 4 and 5, respectively). The shift in the enzyme production pattern observed in culture B3 after ~13 h of induction (Figure 7a) was caused by the decay in specific enzyme activity, as shown in Figure 5, associated to plasmid instability and viability loss. A similar trend can be seen in the productivity curve obtained for culture B3 (Figure 7b).

Process economics is an important factor to be considered when selecting a cultivation strategy and a simple estimate of the costs associated only to the different media formulation, using the same approach described at [46], was performed. The main results, summarized at Table 3, point out the intermittent fed-batch auto-induction protocol (B1) as the most cost-effective cultivation strategy, thanks to its low media cost. Although the continuous fed-batch strategy based on complex medium (B2) led to the highest volumetric enzyme activity, superior production costs related to the large amount of reagents required to achieve and maintain high cell densities restricted its economic performance. Concerning the fed-batch strategy based on defined medium (B3), the 3.4 fold increase of enzyme cost is mainly due to the lower volumetric enzyme activity achieved at this operation mode, since the cost of the culture medium is similar to B2.

**Table 3:** Total cost associated to media used at B1, B2 and B3 cultures and the corresponding estimate of the cost per unit of PGA produced

**Culture ID**	**Batch ($)**	**Pulse ($)**	**Feed ($)**	**Total ($)**	**Total volume (L)**	**Medium cost (per L)**	**Vol Enz Act (U L**^**-1**^**)**	**Enzyme cost ($ U**^**-1**^**)**
B1	69.30	21.50	---	90.81	4.2	21.62	94000	0.00023
B2	55.23	16.11	321.06	392.40	5.6	70.10	208222	0.00034
B3	52.58	71.10	254.36	378.04	5.4	70.00	89000	0.00079

### Comparison of PGA expression for different clones of recombinant *E. coli*

The most recent and relevant results reported for PGA expression using different clones of *E. coli* and different induction strategies are presented in Table 4. Due to the high cell concentration reached, the volumetric enzyme activity and productivity obtained in this work were superior to all the values reported using *rE. coli,* in the literature to date. The specific PGA activity (HCDC and auto-induction) was higher than the best published values [6,33]. These results demonstrate the importance of identifying cultivation conditions that can contribute to the twin goals of increased biomass formation and enhanced gene expression. In the present study, the use of a low induction temperature allied to a fed-batch cultivation strategy using complex medium was crucial for achievement of a superior enzyme titer and maximization of productivity.

**Table 4:** **PGA production in various *E. coli *** **expression systems for bioreactor cultures carried out under different induction strategies**

***E. coli *** **strain/construction**	**Ind.**	**Op. mode**	**T**_**ind **_**(°C)**	**[Biomass] (gDCW L**^**-1**^**)**	**EA**_**sp **_**(IU DCW**^**-1**^**)**	**Vol. Enz.Act. (IU mL**^**-1**^**)**	**Product. (IU mL**^**-1**^ **h**^**-1**^**)**	**Ref.**
RE3/ pKA18	Phenyl acetic acid	Batch	28	5	1000	4.5	0.28*	[33]
MDDP7/ pTrcKnPAC2902	IPTG	Fed-batch	28	33	1020*	37.5	0.48*	[6]
W3110/pPA102	Cheese whey	Batch	29	1.5	781	0.901	0.053*	[23]
9633/pGL-5	IPTG	Fed-batch	30	96.9*	157*	15	0.63*	[24]
BL21(DE3)/pET28b	IPTG	Batch	28	12	800	10	0.42*	[21]
DH5α/ Psmlfpga	IPTG	Batch	30	21.7*	153 mg gDCW^-1^	79.88	0.67*	[22]
DH5α/pET30b	Constitutive	Batch	28.5	4.5	NA	43	2.58*	[20]
BL21(DE3)/pT101/D-TOPO	Lactose	Continuous fed-batch	20	100	2000	190	7.8	This work
BL21(DE3)/pT101/D-TOPO	Lactose	Intermittent fed-batch	20	38	2800	94	3.9	This work

*Estimated from reported data.

*NR* Not reported.

## Conclusions

High enzyme concentration (208222 IU L^-1^) and productivity (6941 IU L^-1^ h^-1^) were achieved for the continuous fed-batch culture with complex medium. Using the auto-induction strategy, both enzyme productivity and concentration can be limited by the accumulation of biomass that may occur in this operational mode. Nevertheless, it remains a very attractive approach, because an enzyme extract with high PGA activity (130 IU per mL of centrifuged cell lysate) can be obtained in just 15 h of simple batch culture, even without an additional pulse of lactose. The results obtained indicate that the innovative high cell density strategy used in this work, combining exponential glycerol feeding, an intermittent lactose supply, and a low induction temperature (20°C), is a promising technique for on-demand penicillin G acylase production by recombinant *E. coli* cells at lower production cost.

## Methods

### Microorganism

*Escherichia coli* BL21 (DE3) was donated by the Laboratory of Biochemistry, Department of Physiological Sciences, UFSCar, São Carlos, Brazil. The pT101/D-TOPO plasmid with the *pac* gene from *E. coli* ATCC11105 responsible for PGA production [47,48] was kindly provided by the Laboratory of Biocatalysis, ICP-CSIC, Madrid, Spain. The transformation of *E. coli* was performed by heat shock in the presence of CaCl_2_.

### Culture media

Shake flask cultivations were carried out using LB medium containing tryptone (10 g L^-1^), yeast extract (5 g L^-1^), sodium chloride (10 g L^-1^), and ampicillin (100 μg mL^-1^), at pH 7. For bioreactor cultures, intermittent fed-batch B1 was performed using auto-induction medium (adapted from Studier and Silva et al. [27,29]) with pulse supplementation. Continuous fed-batch cultures B2 and B3 were carried out using complex medium [29] and defined medium [26], respectively. The compositions of all the media used are provided in Table 1. Ampicillin (150 μg mL^-1^), 30% propylene glycol antifoaming agent (1 mL L^-1^), and a metal solution [29] were added to the media used for the bioreactor cultivations.

### Analytical methods

#### Cell concentration

During the bioreactor cultivations, the cell concentration was monitored using measurements of the optical density (OD) of the culture broth (λ = 600 nm), dry cell weight (gDCW L^-1^) determinations, and viable cell counts (c.f.u. mL^-1^), as well as by on-line recording of broth permittivity (pF cm^-1^) using a biomass sensor [26]. In the shake flask experiments, the cell concentration (C_X_) was related to the optical density using the expression:

(1) $$C_{X}\left(\mathrm{gDCW}\;L^{‒1}\right)=\left(0.49\pm 0.01\right)*\left(OD_{600\;\mathrm{nm}}\right)$$

#### Plasmid stability and cell viability

Diluted samples of culture broth were spread aseptically onto plates of LB agar, in some cases supplemented with ampicillin, and incubated for 16 h at 37°C. Cell viability and plasmid stability were evaluated by counting the final number of colonies formed [30].

#### Enzymatic activity and related variables

Samples were centrifuged at 10,000 × g for 5 min at 4°C, re-suspended in 50 mM phosphate buffer (pH 7.5), and disrupted by sonication using 6 pulses of 30 s (8-10 watts) at 20 mHz, with intervals of 20 s. After further centrifugation at 14,000 × g for 20 min at 4°C, the supernatant was collected and used for analyses of enzymatic activity and total soluble protein concentration (Bradford method [49]). In addition, SDS-PAGE [50] was employed to identify the presence of inactive pre-pro-PA and the α and β PGA subunits in the supernatant and pellet derived from the cell lyses.

PGA activity was determined by hydrolysis of a chromogenic substrate, 1 mM 6-nitro-3-(phenylacetamido)benzoic acid (NIPAB), in 0.1 M phosphate buffer (pH 7.5) at 37°C, according to the method described by Kutzbach and Rauenbus [51]. A calibration curve (Equation (2)) was prepared to further convert PGA activity (in U_NIPAB_ mL^-1^) to the equivalent penicillin G activity (in IU mL^-1^). The enzymatic reaction product (6-APA) was quantified using the colorimetric method described by Balasingham et al. [52].

(2) $$EA_{\mathrm{penG}}=\left(2.2\pm 0.1\right)*EA_{\mathrm{NIPAB}}$$

The specific enzyme activities were expressed in terms of the total soluble protein content (EA_sp_Prot_) and the cell mass (EA_sp_Biom_) using Equations (3) and (4), respectively. The corresponding enzyme concentrations (C_enz_) and productivities (Pr_enz_) per volume of cultivation medium were calculated using Equations (5) and (6), respectively. In these expressions, EA (IU mL^-1^) is the enzyme activity obtained from Equation (2), C_X_sonic_ (gDCW L^-1^) is the biomass concentration in the disrupted cell suspension, C_X_ (gDCW L^-1^) is the biomass concentration in the cultivation broth, and Δt is the elapsed cultivation time.

(3) $$EA_{\mathrm{sp}\_\mathrm{Pr}\mathrm{ot}}\left(U\cdot m{g_{\mathrm{prot}}}^{-1}\right)=\frac{\mathrm{EA}}{C_{\mathrm{sol}\_\mathrm{prot}}}$$

(4) $$EA_{\mathrm{sp}\_\mathrm{Biom}}\left(U\cdot \mathrm{gDC}W^{-1}\right)=\frac{\mathrm{EA}\cdot 1000}{C_{X\_\mathrm{sonic}}}$$

(5) $$C_{\mathrm{enz}}\left(U\cdot L^{-1}\right)=EA_{\mathrm{sp}\_\mathrm{Biom}}\cdot C_{X}$$

(6) $${\mathrm{Pr}}_{\mathrm{enz}}\left(U\cdot L^{-1}\cdot h^{-1}\right)=\frac{C_{\mathrm{enz}}}{\mathrm{\Delta t}}$$

#### Concentrations of organic acids

The concentrations of organic acids were determined by HPLC, with UV detection at 210 nm. The stationary phase was an Aminex HPX-87H column (maintained at 50°C) and the mobile phase was 5 mM sulfuric acid, at a flow rate of 0.6 mL min^-1^.

#### Partial purification of recombinant PGA

The purification procedure followed the protocol developed by Pinotti et al. [44]. In brief, the biomass harvested at the end of the cultivations in defined or complex media was disrupted by sonication in 20 mM citrate buffer at pH 4.8 (used as the adsorption buffer) and centrifuged at 7000 × g for 10 min to obtain the crude soluble enzyme extract. PGA adsorption was carried out using Streamline SP cation exchange resin. The desorption buffer employed for enzyme elution was 150 mM NaCl. The parameters η^process^ (overall yield) and PF (purification factor) were used to compare the performance of the purification process for the different enzyme extracts. Full details of the calculation procedures can be found in Pinotti et al. [44].

### Experimental procedure

Preliminary shake flask cultures were conducted to assess the influence of induction phase temperature on enzyme production. Five conditions were investigated (in triplicate), with temperatures ranging from 18 to 28°C. Intermittent fed-batch (B1) and continuous fed-batch (B2 and B3) bioreactor cultures were carried out using the selected induction temperature and different induction strategies.

#### Shake flask cultures

The shake flask cultivations were carried out for ~30 h using 1 L flasks containing 100 mL of LB medium, incubated at 37°C with agitation at 250 rpm during the initial growth phase. Once the OD_600_ of the culture reached 1.5, the flasks were transferred to another incubator kept at the desired temperature (18, 20, 22, 24 or 28°C), and induction was performed by adding a pulse of 0.25 mM IPTG.

#### Bioreactor cultures

A 300 mL aliquot of exponentially-growing cell suspension (OD_600_ ~2.0) was transferred to a 5 L laboratory bioreactor containing 3.5 L of the complex or defined auto-induction media described in Table 1. All cultivations were monitored using SuperSys_HCDC^R^[26,53]. The temperature was maintained at 37°C during the growth phase, and reduced to 20°C prior to induction. The pH was maintained at 6.9 by addition of 28% (w/v) NH_4_OH solution. The dissolved oxygen concentration was controlled at 30% of saturation by automatically adjusting both the agitation speed (in the range 200-600 rpm) and the composition of the gas stream supplied to the bioreactor (by mixing pure oxygen with air). The total inlet gas flow rate was maintained at 5 L min^-1^ using two mass flow controllers. The broth permittivity was monitored using a biomass sensor (Fogale Nanotech), as described by Horta et al. [26,53].

For the continuous fed-batch cultures (B2 and B3), the exponential feed flow was initiated once the carbon sources in the batch medium had been exhausted. The feed flow rates of the complex (culture B2) or defined (culture B3) media were calculated using Equation (7), which assumes that the growth rate is only dependent on the limiting substrate [54], and were automatically controlled by the supervisory system [26].

(7) $$F=\left(\frac{\mu }{Y_{x/s}}+m\right)\cdot \frac{C_{\mathrm{xo}}-V_{o}}{C_{\mathrm{SF}}-C_{\mathrm{SR}}}\cdot e^{\left(\mu_{\mathrm{set}}-t\right)}$$

In Equation (7), F (L h^-1^) is the feed flow rate, μ (h^-1^) is the actual specific growth rate, μ_SET_ (h^-1^) is the desired specific growth rate, Y_X/S_ (gDCW g_substrate_^-1^) is the biomass yield coefficient, m (h^-1^) is the maintenance coefficient, C_X0_ (gDCW L^-1^) and V_0_ (L) correspond to the cell concentration and volume, respectively, at the beginning of the fed-batch phase, C_SF_ (g_substrate_ L^-1^) is the glycerol concentration in the supplementary medium (Table 1), C_SR_ (g_substrate_ L^-1^) is the expected residual concentration of glycerol in the reactor, and t (h) is the elapsed feeding time. The parameters Y_X/S_ and m were estimated from the previous batch data, and are included in Table 5. Analogously, C_SR_ was set to 5 g L^-1^, based on information obtained in earlier experiments [29].

**Table 5:** Parameter values used for setting up the feeding flow rate (Eq. 7) for fed-batch bioreactor cultures B2 (complex medium) and B3 (defined medium)

**Parameter**	**B2**	**B3**
m (h^-1^)	0.001	0.05
Y_X/S_ (gDCW g_substrate_^-1^)	0.717	0.485

The exponential feeding profile was regulated using SuperSys_HCDC^R^, which automatically re-tuned the parameter μ_set_ every 10 min, using the values of μ inferred from on-line permittivity measurements provided by the capacitance biomass sensor [26].

In intermittent fed-batch culture B1, additional glycerol and lactose were supplied as a pulse (Table 1) when the OD_600_ value reached ~70, in order to provide enough inducer and carbon source for the prolonged induction phase, which lasted 24 h. In continuous fed-batch cultivations B2 and B3, the induction phase was started when the OD_600_ reached ~100, and also lasted 24 h. For experiment B3 (defined medium), the induction was performed by adding an IPTG pulse (1 mM final concentration). For experiment B2 (complex medium), induction was carried out by adding multiple pulses of 400 mL of lactose solution (200 g L^-1^). In all bioreactor cultures, ampicillin pulses were applied every 6 h after induction to maintain selective pressure on the cells.

## Competing interests

The authors declare that they have no competing interests.

## Authors’ contributions

AMV participated in the performance of all the experiments and drafted the manuscript. AJS was involved in revising the manuscript critically for important intellectual content. ACLH implemented the control and instrumentation tool used to supervise the bioreactor experiments. CRS helped to perform the continuous fed-batch experiments. GC and GGS helped to perform the auto-induction experiment. RLCG helped in discussion of the purification results. TCZ conceived the study and helped to draft the manuscript. All authors read and approved the final manuscript.
